# Deep locomotion prediction learning over biosensors, ambient sensors, and computer vision

**DOI:** 10.1371/journal.pone.0342793

**Published:** 2026-02-23

**Authors:** Madiha Javeed, Ahmad Jalal, Dina Abdulaziz AlHammadi, Bumshik Lee

**Affiliations:** 1 Faculty of Computer Science, Preston University, Islamabad, Pakistan; 2 Department of Computer Science, Air University, Islamabad, Pakistan; 3 Department of Computer Science and Engineering, College of Informatics, Korea University, Seoul, Korea; 4 Department of Information Systems, College of Computer and Information Sciences, Princess Nourah bint Abdulrahman University, Riyadh, Saudi Arabia; 5 Energy AI, Korea Institute of Energy Technology (KENTECH), Naju, Korea; Polytechnic University of Marche: Universita Politecnica delle Marche, ITALY

## Abstract

Innovative technologies for developing intelligent systems related to locomotion prediction learning are crucial in today’s world. Human locomotion involves various complex concepts that must be addressed to enable accurate prediction through learning mechanisms. Our proposed system focuses on locomotion learning through vision RGB devices, ambient sensors-based signals, and physiological motions from biosensing devices. First, the data is acquired from five different scenarios-based datasets. Then, we pre-process the data to mitigate the noise from biosensors and extract body landmarks and key points from computer vision-based signals. The data is then segmented using a data windowing technique. Various features are extracted through multiple combinations of feature extraction methodologies, followed by feature reduction using optimization techniques. In contrast to existing systems, we employ both machine learning and deep learning classifiers for locomotion prediction, utilizing a modified body-specific sensor-based Hidden Markov Model and a deep Exponential Residual Neural Network, respectively. System ontology is also presented to elucidate the relationships among the data, concepts, and objects within the system. Experimental results indicate that our proposed biosensor-based system exhibits significant potential for effective locomotion prediction learning.

## Introduction

Human locomotion learning is an important aspect of artificial intelligence (AI)-based systems for human motion applications [1]. Intelligent sensors-based system processing has given a boost to the locomotion prediction learning field [2–4]. It is beneficial to utilize advanced sensory devices, analyze those signals, and enable human motion pattern recognition in healthcare systems, smart homes, lifelog routine management, and smart surveillance systems [5–8]. Biosensors such as inertial measurement units (IMU) and electromyography (EMG) sensors can acquire physiological data important for human body dynamics exploration [9]. Algorithms, including machine learning and deep learning, can process this data to predict different motion patterns, including gait, postures, and movement [10].

Ontology agents are the AI-relevant agents used to enhance the ability of a system to process and interpret information. An ontology can support representing the knowledge related to the locomotion prediction system domain. It contains the data interrelationships, concepts, and characteristics to provide a structured framework for agents to share and integrate information, which will help make more informed decisions [11,12]. Since our proposed system consists of multiple sensors-based data, ontology will facilitate understanding and incorporating different sensors to enhance the AI reasoning of the agents, as well as learning and communication abilities [13]. Ontological agents can help adapt new locomotion activities and update knowledge to make our proposed system adaptive. They can also enhance the system’s ability to predict and respond to changes in motion patterns [14].

Several systems have been proposed in this research area to predict human locomotion using sensor data. While some studies rely on single sensors [15–17], others integrate multi-sensors, proposing multi-modal systems [18–22]. However, these approaches face challenges such as signal drift [15], data fusion problems [18], background noise present in biosensors-based data [21], pre-processing step missing [20], sensors-based calibration limitations [22], inability to distinguish different actions [22], features extraction and selection not applied [23], limited data [16,24], irrelevant descriptors [20,23], and restricted movements recognition [17] causing degraded performance when it comes to locomotion prediction learning [23–25].

To address these limitations, we propose an intelligent system that integrates biosensors, ambient sensors, and computer vision for efficient and effective human locomotion prediction learning by using multi-sensory devices instead of single sensor. First, pre-processing is performed for noise reduction and a novel kinematic and static patterns recognition approach is defined in biosensors-based signals along with body-point extraction from videos explained in section 3.2. This component has helped in signal drift, sensors-based calibration issues and background noise reduction present in traditional systems. Next, a data segmentation method is utilized explained in detail in section 3.3, which supported in achieving better results in terms of system performance by reducing the overall data size of monitoring system and dividing it into segments for efficient processing. It helped in addressing the data fusion problem reduction, which is partly caused by the big sized data processing. Also, data fusion has been performed at the features level to resolve challenges faced for multi-sensory devices-based data integration in previously proposed systems. Then, relevant descriptors are extracted from each type of sensor for catering distinguished patterns in each human action explained in section 3.4, followed by the reduction of extracted features to handle the dimensionality issue present in conventional approaches given in section 3.5. Furthermore, our proposed system recognizes various human actions captured during data collection using multiple scenario-based datasets mitigating the limited data challenge present in literature. There are different types of actions that can be performed in different scenarios of lifelog routine. The conventional systems have focused over limited scenarios that makes the system’s practical implementation very limited but our proposed system has focused over wide range of movements recognition explained in section 4.1. However, by experimenting in different setups-based data, this system has the ability to learn multiple scenarios and supports human locomotion prediction learning, yielding acceptable results causing performance enhancement.

This paper is organized as follows: Section 2 gives a detailed overview of our proposed system and its implementation details. Section 3 shows the experimental results and their outcomes for each sensor type and the complete system. Section 4 discusses the limitations and challenges present in the proposed method. Section 5 concludes the paper with future directions.

## Methods

**Fig 1:**
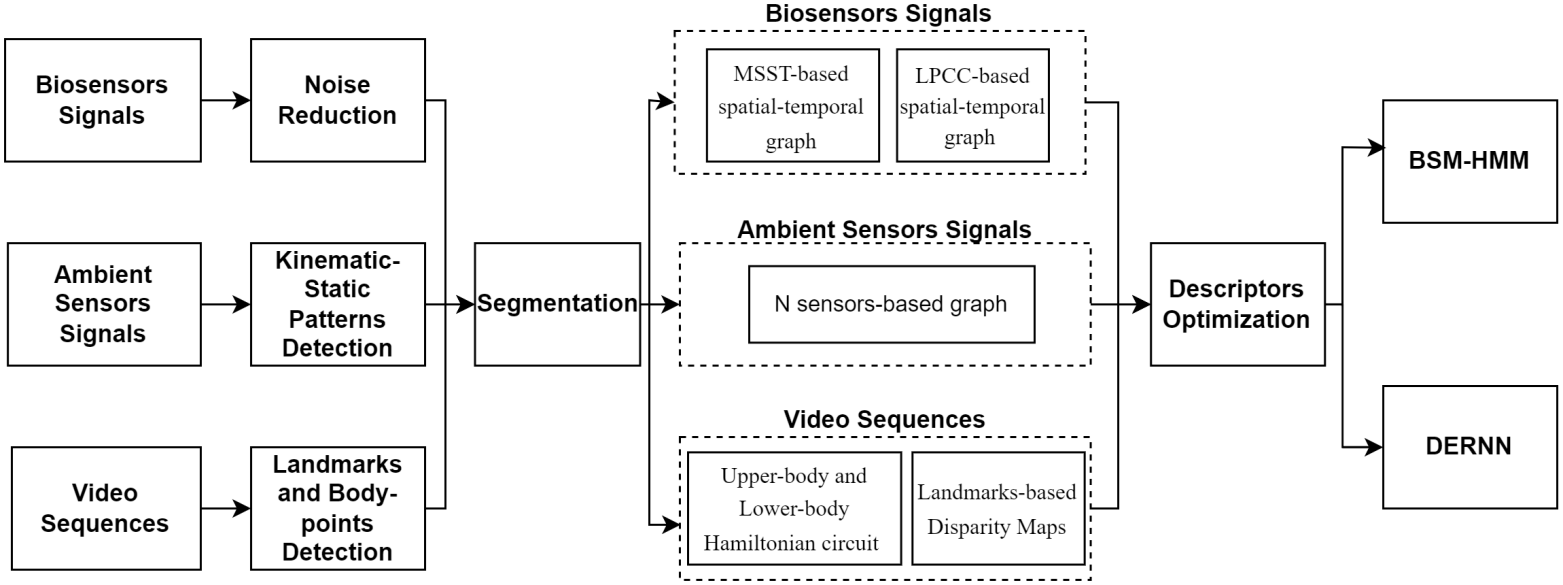
Comprehensive framework of our proposed deep adaptive locomotion prediction learning system over ontology agents and multi-sensory devices where the signals from multi-sensors have been first pre-processed. Next, data segmentation has been applied followed by features extraction for each type of sensors-based data. After fusing the different extracted features, descriptors have been optimized and finally for locomotion classification, BSM-HMM and DERNN have been used.

This section provides a comprehensive framework detail for our proposed locomotion prediction system. It provides a detailed system overview, from data acquisition to locomotion prediction both via machine learning and deep learning algorithms. Fig 1 shows the overall architecture of our proposed system.

### Data acquisition

In the proposed method, we acquired data from five different datasets using all three types of sensors, including biosensors, ambient sensors, and vision sensors. Data was collected from Opportunity++ [26], CMU-MMAC [27], Berkeley-MHAD [28], HWU-USP [29], and LARa [30] datasets. The reason for selecting these five datasets is to gather data from different human locomotion to cater to the versatile complex activities and the simple actions performed by humans in daily lifelog routines.

### Pre-processing

**Fig 2:**
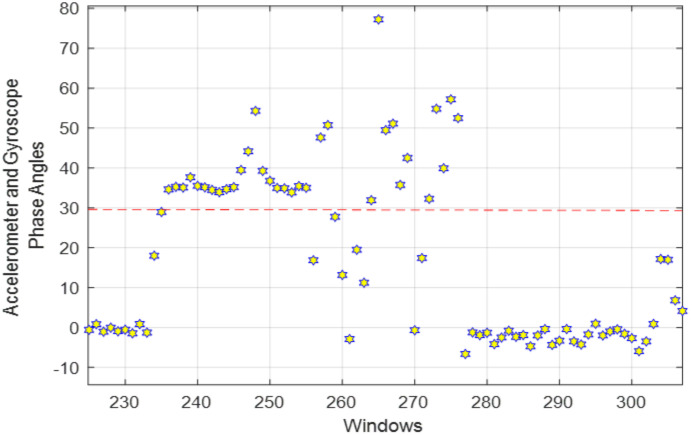
Kinematic and static patterns detected above and below the red dashed threshold (30) line over HWU-USP dataset.

It is an important step in our proposed system for locomotion prediction over multi-sensory devices and ontology agents. The noise present in the signals can cause degraded performance through incorrect pattern recognition. As a pre-processing step, a Wavelet Transform Quaternion-based filter [31] is utilized for biosensor filtration. First, the three signal readings from biosensors, including acceleration, gyroscope, and magnetometer, are retrieved from the IMU. To remove noise, we use the calibration phase to remove gravitational error from acceleration, drift error from the gyroscope, and magnetic error from magnetometer signals. Further, we use Quaternions and a gradient descent technique to normalize the data into vectors in a mapping and optimization phase. After filtration, the kinematic and static patterns [32] are detected from IMU signals to recognize the abrupt changes in signals caused by complex motion signals. The phase angle [33] is used to detect the learning phase of signals using (1) as:


$$J_{w}=\;\theta_{w}+\;\sigma_{w}+\;\partial_{w}$$ (1)

where $\theta_{w}$ is the angle of the w^th^ acceleration signal, $\sigma_{w}$ provides the w^th^ gyroscope signal angle, $\partial_{w}$ shows the angle of the w^th^ magnetometer signal and $J_{w}$ is the selected the w^th^ signal. Fig 2 shows the phase angles extracted from each acceleration, gyroscope, and magnetometer signal over a red threshold line separating the kinematic and static signals. The yellow stars above the threshold show kinematic pattern detection, and the ones below the threshold represent static patterns.

**Fig 3:**
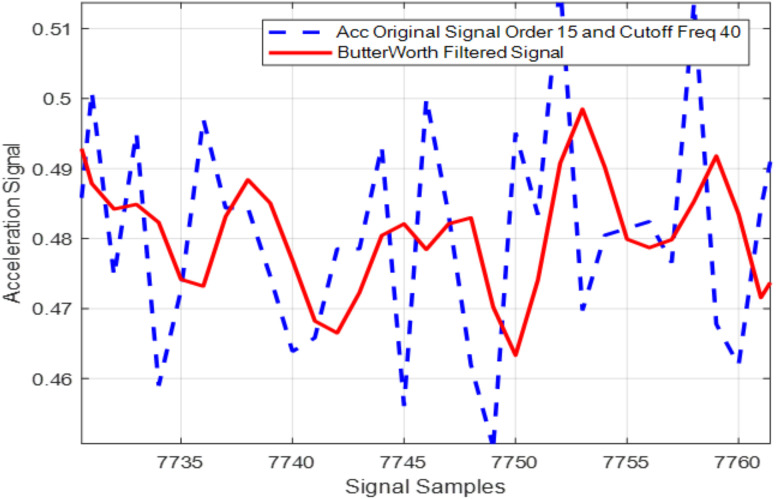
Actual acceleration signal and Butterworth filtered signal over Opportunity++ dataset.

The Butterworth filter [34] is used to pre-process the ambient sensor signals to reduce the surrounding noise. The filtration is performed using (2) as:


$$H(r)=\;\frac{\text{1}}{T^{\text{2}}+\;\sqrt{\text{1}\text{5}}T+\;\text{1}}$$ (2)

where T presents a domain transfer function and for the 15^th^ order Butterworth filter, which helps reduce the noise much better compared to other similar filters. Fig 3 shows the actual acceleration and filtered signals over the Opportunity++ dataset. As illustrated in Fig 3, the filter effectively reduces noise in accelerometer signals from inertial sensors employed for ambient sensing.

**Fig 4:**
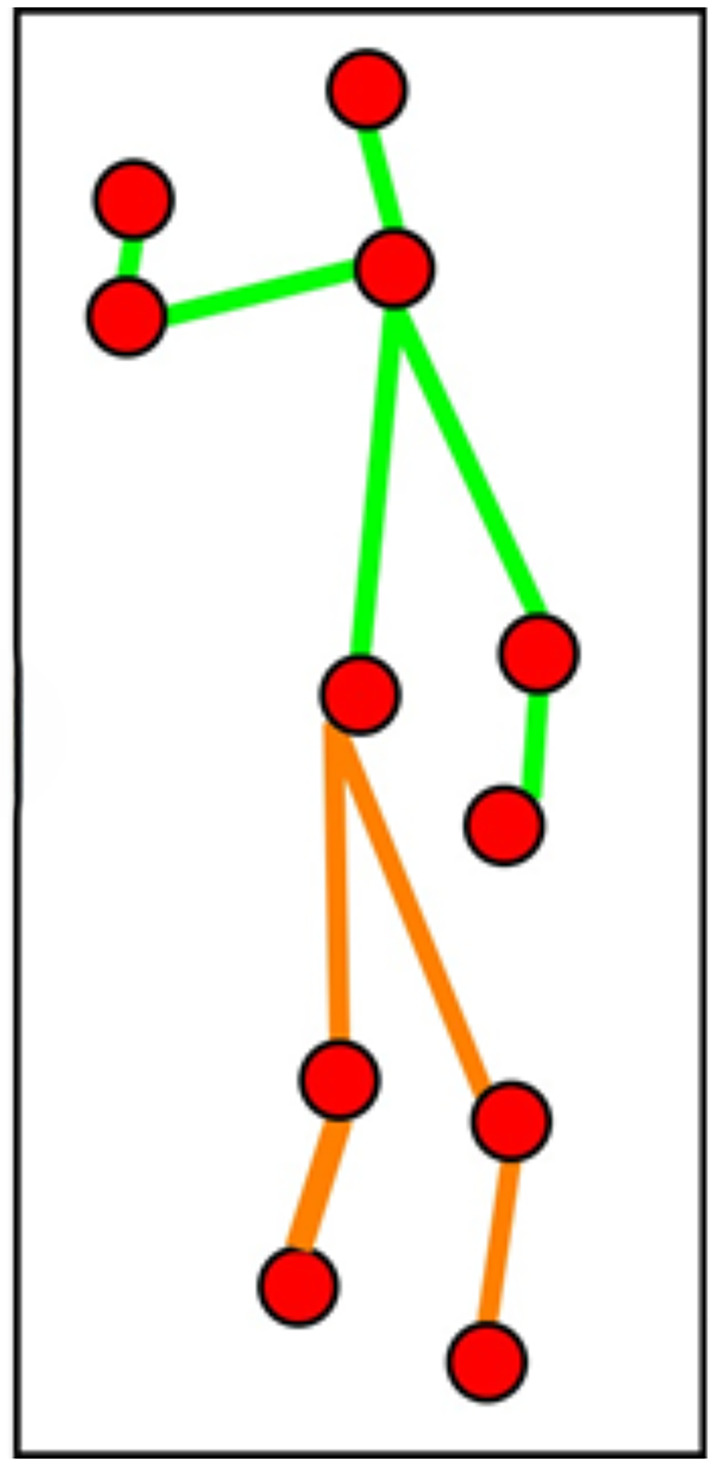
2D stick model after landmarks mining and eleven body-points extraction over Berkeley-MHAD dataset.

To pre-process the computer vision-based video sequences from RGB videos, a delta of 45 images is selected to avoid processing costs and delays in the performance of the locomotion prediction system. A background image is selected for each type of video and subtracted from all the video sequences to detect human figures. Next, a landmark detection method [35] is applied to calculate the human position in a frame *p* as (3):


$$H_{S}^{p}=\left(L_{F}^{p}\;\leftarrow \;L_{F}^{p-\text{1}}+\;\Delta T_{F}^{p-\text{1}}\right)+\;H_{B}^{p}$$ (3)

where $H_{B}^{p}$ is the frame *p*’s boundary and $H_{S}^{p}$ is the human silhouette. To extract the human torso landmark, ${LT}_{g}$ is obtained using (4) as:


$${LT}_{g}=\;\frac{\left|S_{hx}+\;S_{hy}+\;S_{g}\right|}{\sqrt{S^{\text{2}}}}$$ (4)

where $S_{hx}+\;S_{hy}$ presents the addition of human silhouette height and width to extract the human shape pixel g. The midpoint of ${LT}_{g}$ gives the torso mid-point. Now, by utilizing the body shape and size of the human silhouette, the head point and feet H,F landmarks $L_{H,F}^{f}$ are detected as (5):


$$L_{H,F}^{f}\;\leftarrow \;L_{H,F}^{f-\text{1}}+\;\Delta T_{H,F}^{f-\text{1}}$$ (5)

where f is the frame sequence for each dataset, after the detection of head and feet landmarks, the midpoints are used to represent the head and feet body-points. Then, the neck, elbow N,E, and knees are detected using (6):


$$L_{N,E}^{f}=(L_{H,F}^{f}-\;T_{p})/\text{2}$$ (6)

where $L_{N,E}^{f}$ is the landmarks detected for the neck and elbow. After dividing by half, the midpoint of the torso and head provides the neck point, and the midpoints of elbow landmarks give the wrist or hand body-points. Elbow points are tricky and need to be extracted by mining the landmark size and considering one of the most right, most left, lowest, or highest midpoints from elbow landmarks. The knee points are determined by finding the midpoints between the torso and feet. This process aids in constructing a 2D stick model [36] by connecting the extracted midpoints from the human silhouette. Fig 4 shows a 2D stick model after extracting landmarks and body-points. The red dots in Fig 4 give the eleven body-points extracted from each landmark. The red dots are further connected using the green and orange lines, where the green lines indicate the upper body 2D stick model and the orange lines show the lower body 2D stick model.

### Data segmentation

**Fig 5:**
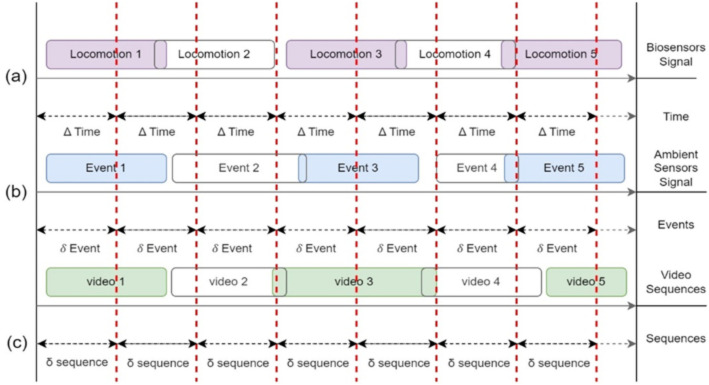
Data segmentation over the three types of sensors-based pre-processed data including biosensors, ambient sensors, and video sequences-based signals.

Comprehensive data segmentation [36] is applied to the pre-processed data obtained from all three types of sensors. Fig 5 shows the data segmentation process performed on a data chunk by incorporating the time, events, and sequences. The red dashed lines indicate the segment separation for each ∆ time, δ event, and ō sequence. Specially, locomotion *n* presents the biosensor signals, event *n* corresponds to the ambient sensor signal, and video *n* denotes the video sequence. Experiments were conducted using window sizes of 2, 3, and 5 seconds over the combined dataset. Based on empirical analysis, we found that 3-second overlapping windows yielded the most efficient and effective results.

### Descriptors extraction

**Fig 6:**
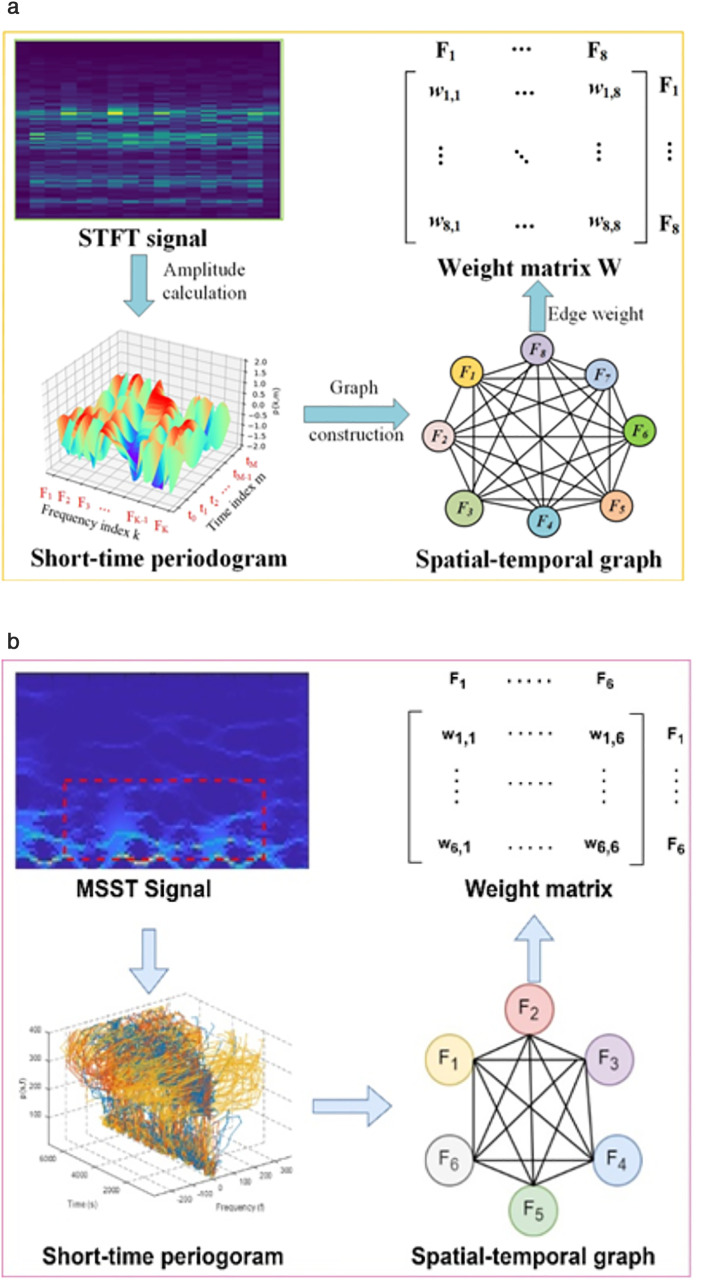
Kinematic Descriptors Extraction: (a) previous study with spatial-temporal graph extraction via STFT [38]; (b) Our proposed spatial-temporal graph extraction through MSST signal shows the kinematic-energy in the dotted box (Red box).

To utilize their characteristics, we propose two novel descriptor extraction methods for each kinematic and static pattern. To do this, a spatial-temporal graph from multi-synchro squeezing transform (MSST) [37] signals are extracted for kinematic patterns using (7) as:


$$Ts^{\left[I\right]}(t,\gamma )=\;\int_{-\infty }^{+\infty }Ts^{I-\text{1}}(t,\gamma )\delta \left(\gamma -\hat{\omega }(t,\omega )\right)d\omega ,$$ (7)

where $Ts^{\left[I\right]}(t,\gamma )$ is the time-frequency spread for the *I*-th iteration. Then, a short time periodogram p(t,f) can be calculated using (8) as:


$$p(t,f)=\frac{\text{1}}{L}\;|\;Y(t,f){|}^{\text{2}}$$ (8)

where p(t,f) is obtained over L window length, for time t and frequency f. Next, six frequencies-based nodes are used to construct a spatial-temporal graph. To get the graph, Laplacian matrix (LM) can be obtained using eigenvalues and eigenvectors as (9):


$$LM=Y\cap Y^{T}$$ (9)

where $Y=[y_{\text{0}},\;\ldots ,\;y_{n-\text{1}}]$ is the eigenvector and $\cap \;=diag[\alpha_{\text{0}},\;\ldots ,\;\alpha_{n-\text{1}}]$ is the matrix for eigenvalues. Fig 6 compares our proposed technique with the previous study [38]. The earlier method used short-time Fourier transform (STFT) signals, while we propose using MSST signals, which are more effective for analyzing impulsive-like signals [39] and better suited for handling the complexity of kinematic energy signals, as shown in Fig 6.

**Fig 7:**
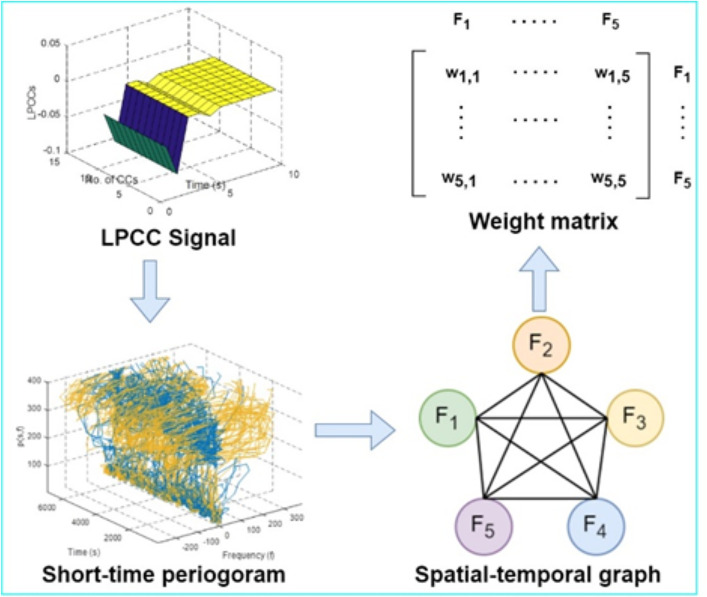
LPCC-based spatio-temporal graph extraction for static patterned biosensor signals by using short-time periogram and weighted matrix.

As a next step, a linear prediction cepstral coefficient (LPCC) based spatial-temporal graph is extracted to extract descriptors from static biosensor signals. Five frequencies are used to transform a short-time periodogram into a spatial-temporal graph. The cepstrum can be extracted using (10) as:


$$\begin{array}{c} e_{m}=\;l_{m}+\;\sum_{j=\text{1}}^{m-\text{1}}\left(\frac{j}{m}\right)e_{k}l_{m-k}\;,\;\text{1}\;\leq m\;\leq p \end{array}$$ (10)

where $e_{m}$ is LPCC, $l_{m}$ is linear prediction coefficient, p offers the number of relevant to LPCC, and j denotes the number of iterations. Fig 7 illustrates our proposed spatial-temporal graph for static biosensor signals via LPCC.

**Fig 8:**
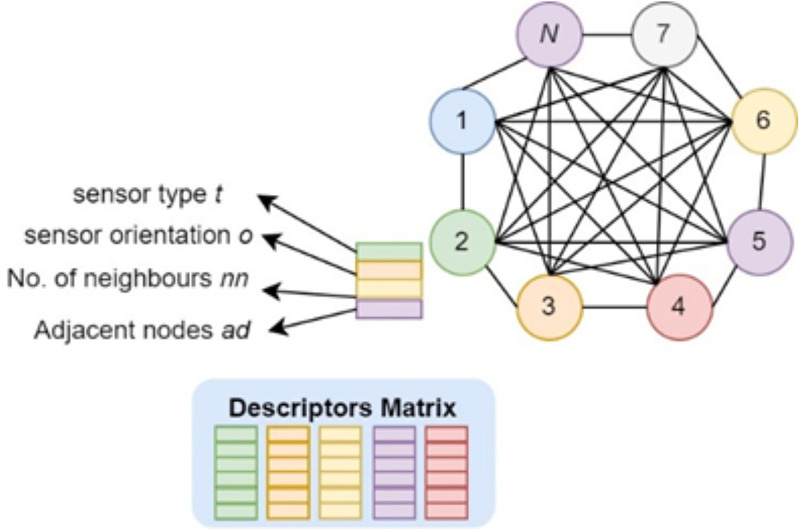
Descriptors extraction from N sensors-based graph using sensor type, sensor orientation, number of neighbours, and adjacent nodes.

To extract descriptors from ambient sensor pre-processed data, we propose a *N*-sensors based graph *F* using descriptors matrix *d* and adjacency matrix *m* [40] as (11):


F=(d,m) (11)

$$\begin{array}{c} d=\sum_{j=\text{0}}^{w}t+nn+o \end{array}$$ (12)

where d is the descriptors, matrix using type t sensors, nn is the number of neighbors, and o orientation for w iterations. Fig. 8 shows the proposed ambient sensor descriptors extraction method in detail. For *N* sensors, we have the fully connected graph as shown in Fig 8. For each sensor, the descriptors are based on sensor type, sensor orientation, number of neighbors, and adjacent nodes, as calculated in (12).

**Fig 9:**
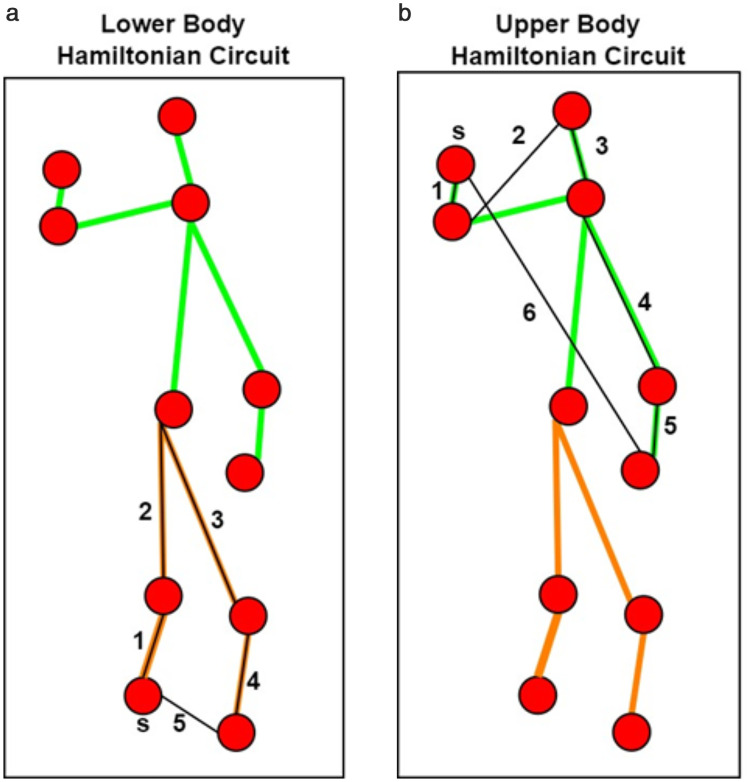
(a) Upper-body Hamiltonian graph; (b) Lower-body Hamiltonian graph over Berkeley-MHAD dataset.

For the video sequences pre-processed data, we utilize the eleven body-points and 2D stick model, including head, neck, left wrist, right wrist, left knee, right knee, left elbow, right elbow, torso, left ankle, and right ankle. A Hamiltonian circuit is used to extract the graph exactly once without edge repetition and returns to the starting node. We have divided the 2D stick model into two Hamiltonian graphs [41], such as the upper body and lower body. Pearson correlation p(i,j) [42] can be calculated for each corresponding node using (13) as:


$$p(i,j)=\;\frac{\sum \left[\left(i_{x}-\overset{―}{i}\right)*(j_{x}-\overset{―}{j})\right]}{\partial i*\;\partial j}$$ (13)

where $\overset{―}{i}$ is the mean for i and $\overset{―}{j}$ is the mean for j, $i_{x}$ is the *x-th* node sample and j is the *y-th* node sample. Afterwards, the descriptors are formed in the matrix shape using nodes and edges, as shown in Fig 9. The red dots in both Fig 9(a) and 9(b) display the eleven body-points extracted. The green lines in Fig 9(a) represent the upper body along with the captioned black Hamiltonian path for the upper body Hamiltonian circuit generated. Fig 9(b) shows the orange lines and the captioned black Hamiltonian path for the lower body Hamiltonian circuit produced.

To get the full body-based descriptors, the disparity $D\left(q_{\text{0}},q_{\text{1}}\right)$ for each two consecutive frame sequences $q_{\text{0}}$ and $q_{\text{1}}$ is calculated using (14) as:


$$\begin{array}{c} D\left(q_{\text{0}},q_{\text{1}}\right)=\;\sum_{x=\text{1}}^{N}\sum_{y=\text{1}}^{M}(q_{xy}^{\text{0}}-q_{xy}^{\text{1}}{)}^{\text{2}} \end{array}$$ (14)

where x and y are coordinates, N, M are the size of the sequences $q_{\text{0}}$ and $q_{\text{1}}$, D is the sum of squared differences, and $L_{x}$ represents a landmark for x coordinate. Next, a landmarks-based disparity map DM(x,y) is calculated using (15) for x and y coordinates. Matching pixels from frame sequences $F_{L}$ and $F_{R}$ are extracted using the sum of absolute values $SAV\left(F_{L},\;F_{R}\right)$ as (16):


$$DM(x,y)=(L_{\tilde{x}}-L_{x})$$ (15)

$$\begin{array}{c} SAV\left(F_{L},\;F_{R}\right)=\;\sum_{i=\text{1}}^{M}\sum_{j=\text{1}}^{N}|\;F_{ij}^{L}-F_{ij}^{R}|\; \end{array}$$ (16)

**Fig 10:**
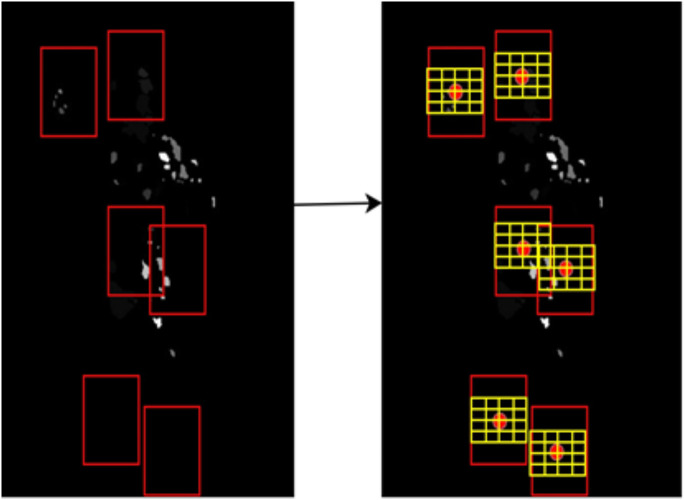
Full body-based disparity map descriptors extraction for Berkeley-MHAD dataset through red dots describing center points used to mine yellow grid giving the 8x8 grid of 4x4 pixels each.

Finally, to calculate the landmarks-based disparity map [43], an 8 × 8 grid g of 4 × 4 pixels each is mined using center point c as (17):


$$\begin{array}{c} g=\;\sum_{i=\text{1}}^{K}\sum_{j=\text{1}}^{L}c\cdot k \end{array}$$ (17)

where c is the center point in landmark L and pixels K. A descriptor matrix can be obtained using full body image sequences as in Fig 10. The extracted landmarks are shown in Fig.10(a), the landmark-based disparity map is calculated using (15) and displayed in Fig 10(b), and the grid is computed using (17) and given in Fig 10(c), where the red dot in each grid denotes the center point c.

### Descriptors selection

After the multi-sensors-based descriptors extraction, data fusion has been applied using the time series. We have applied it to feature-level and descriptors from all three types of sensors have been fused together using feature-level fusion over time. Furthermore, a modified multi-layer sequential forward selection (MLSFS) [44] method is used to reduce the dimensions of extracted descriptors for descriptor selection in the proposed method. Sequential forward selection is utilized to modify this algorithm to achieve reduced vector R using (18) as:


$$R={argmax}_{S_{d}}G(S_{d},\;D,\;M)$$ (18)

where $S_{d}$ is a subset of descriptors of size d selected from the original descriptor set, D represents the dataset containing the input values, M is the classification model used to evaluate the descriptor subset, and $G(S_{d},\;D,\;M)$denotes an evaluation function (e.g., classification accuracy or another performance metric) used to score each subset $S_{d}$ given the dataset D and model M. This equation helps to repeat the selection of descriptors until all the correlations are compared and the final descriptors vector is selected. We have experimented using different types of optimization and selection methodologies and found MLSFS to outperform other techniques, including linear discriminant analysis, Fisher linear discriminant analysis, and sequential forward selection.

### Sensors-based ontology

Due to the large vector size causing heterogeneity even after descriptors reduction, it becomes difficult to manage data from multi-sensory devices. Hence, the domain knowledge in the form of sensors-based ontology [45] is presented in this proposed system. This sensors-based ontology supports the system for better locomotion prediction by explaining the sensors used, interpretations, processes, and characteristics. We have divided the sensors domain into biosensors, ambient, and vision. Then, the interactions with events, time, situation, and network are also presented in the form of strategies. The following equation helps to extract the semantic similarity between two concepts $SS\;\left({con}_{\text{1}},\;{con}_{\text{2}}\right)$ as (19):


$$SS\;\left({con}_{\text{1}},\;{con}_{\text{2}}\right)=\;\frac{\partial \left(d\left({con}_{\text{1}}\right)+d\left({con}_{\text{2}}\right)\right)+\;\theta }{D({con}_{\text{1}},\;{con}_{\text{2}}{)}^{\text{2}}+\;\partial \left(d\left({con}_{\text{1}}\right)+d\left({con}_{\text{2}}\right)\right)+\;\theta }$$ (19)

where $d\left({con}_{\text{1}}\right)$ is the depth of the sememe concept ${con}_{\text{1}}$ with adjustable parameters θ and ∂ and length of path from concept ${con}_{\text{1}}$ to concept ${con}_{\text{2}}$ as $D({con}_{\text{1}},\;{con}_{\text{2}}{)}^{\text{2}}$. We define structural similarity calculation rules as:

1. Parent nodes for concepts and concepts are alike in the constructed ontology tree.
2. Two or more concepts and their children’s nodes are alike.
3. If two concept nodes are alike, then their sibling nodes are also alike.

**Fig 11:**
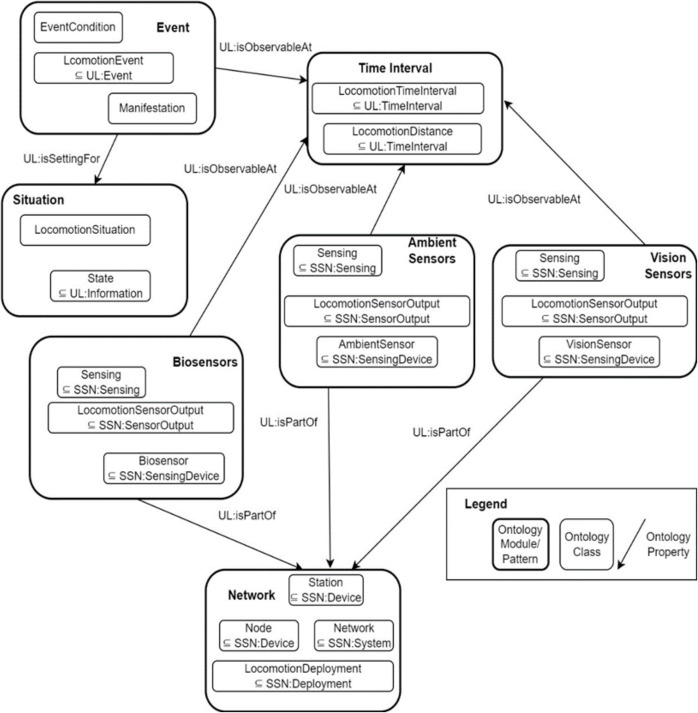
Sensors-based ontology for proposed locomotion prediction system using Event, Situation, Biosensors, Ambient sensors, Computer Vision sensors, and Network concepts.

After defining these rules, we calculate the structural similarity ${Sim}_{x}$ in two concepts as in these equations:


$${Sim}_{x}\left(K\left(i_{\text{1}}\right)\;,K\left(i_{\text{2}}\right)\right)=\;\frac{P(K\left(i_{\text{1}}\right),\;K\left(i_{\text{2}}\right))}{P\left(K\left(i_{\text{1}}\right),\;K\left(i_{\text{2}}\right)\right)+\;P\left(\overset{―}{K\left(i_{\text{1}}\right)},\;K\left(i_{\text{2}}\right)\right)+\;P(K\left(i_{\text{1}}\right),\;\overset{―}{K\left(i_{\text{2}}\right)})}$$ (20)

where $i_{\text{1}}$ and $i_{\text{2}}\;$represent the concepts of different ontologies, $K\left(i_{\text{1}}\right)$ provides the collection of nodes related to the $i_{\text{1}}$ concept [46]. Fig 11 shows the sensors-based ontology proposed for the locomotion prediction system. It contains seven ontological modules or patterns: event, time interval, situation, biosensors, ambient sensors, vision sensors, and network. Each ontology module is related to a few other concepts using the ontology property. Each ontology pattern also consists of a set of ontology classes.

### Locomotion prediction

Machine learning and deep learning each have unique characteristics, and both can be applied to a wide range of applications. However, when it comes to matters involving human life, it is crucial for our system to achieve the best possible results. To this end, we propose a custom machine learning algorithm named Body-specific Sensors Modified based on the Hidden Markov Model (BSM-HMM) and a deep learning model called the Deep Exponential Residual Neural Network (DERNN).

BSM-HMM is inspired by a statistical model [47] consisting of finite states $f_{t}$ at time t, set of vertices j, and a transition probability matrix r(i,j) as hidden Markov model (HMM) M in (21) and (22) as:


$$r(i,j)=prob\left(f_{t+\text{1}}=j\;\right|f_{t}=j)$$ (21)

M=(L,r)    (22)

The probability of visiting a state sequence with events E, possible events M, and parameters ∂ can be extracted using (23).

**Fig 12:**
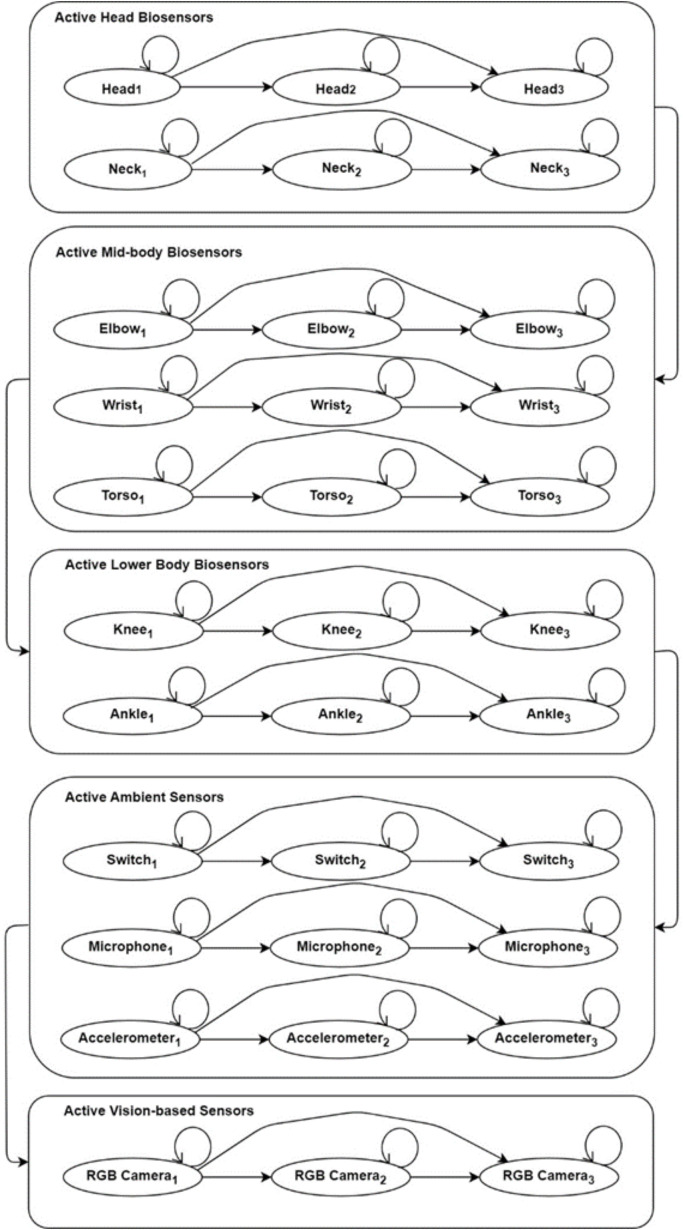
Flow diagram for the proposed BSM-HMM using biosensors, ambient sensors, and computer vision sensors.

An HMM was trained for each kinematic and static patterned signal related to every single dataset. Fig 12 shows the BSM-HMM flow diagram for different body-specific sensors. We separated the sensors-specific HMMs into five individual HMMs using the head, mid-body, lower body, ambient and vision-based specific sensors. Active head-specific sensors-based HMM consists of all the biosensors sensors actively working at the head or neck positions. Next, we have active mid-body biosensors, specifically HMM, representing biosensors attached to the shoulders and waist of the human body. Then, active lower-body specific sensors include all the biosensors attached from the thighs to the feet of the human body. Furthermore, active ambient sensors-specific HMM refers to all the sensors attached to the surroundings of the human, including accelerometers, RFIDs, and PIR sensors-based data classification. Finally, the active vision-based sensors-specific HMM represents the RGB camera extracted data-based HMM, focusing solely on classifying vision data.

**Fig 13:**
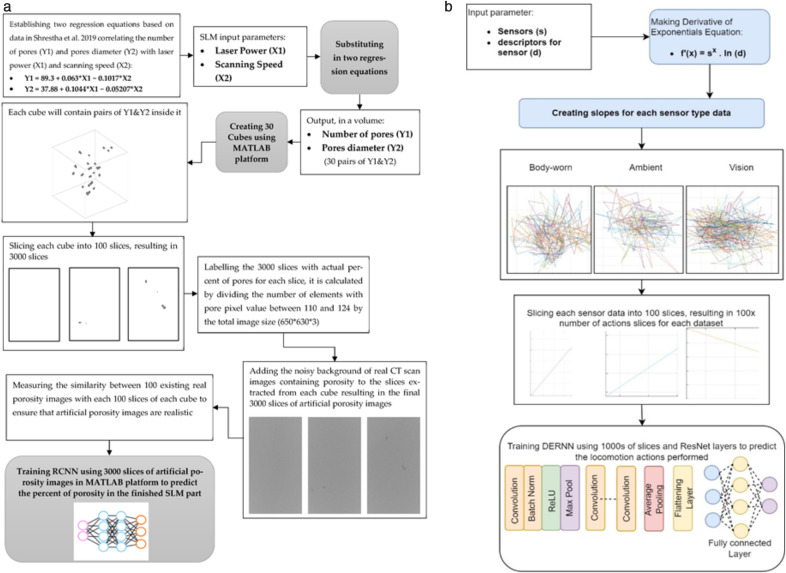
(a) Previous method using regression equations and RCNN for prediction of porosity in computed tomography [48]; (b) Our proposed slopes-based method for each sensor type and DERNN for locomotion prediction.

A deep learning-based model named DERNN is proposed in this study extracted from regression convolutional neural network (RCNN). In [48], a system is proposed for the prediction of porosity in computed tomography (CT) scans using RCNN. We modified the RCNN to cater to the multi-sensory devices-based data requiring multiple sensors. The proposed DERNN can be defined using *n* descriptors for *s* sensors in derivatives of exponential $d^{\prime }(x)$ as (23).


$$d^{\prime }(x)=s\cdot ln(n)$$ (23)

where *p* is a tunable parameter specific to the s-th sensor, s represents the total number of sensors, and n denotes the number of descriptors extracted from each sensor. For each locomotion action performed, different slope values are computed per sensor. Subsequently, each sensor-based slope is divided into 100 segments (or slices) and fed into a residual neural network (ResNet) based on the ResNet-50 framework. A matrix is extracted for each slice and processed by ResNet-50 in five stages. The first stage focuses on multiple layers, such as convolutional, normalization, ReLU, and max pooling. Then, the 2-nd to 5-th stages is the repetition of convolutional layers having input to neurons followed by a pooling layer to reduce the descriptors. Further, a flattening layer is utilized to change the matrix from a 2D to a 1D constant linear vector. It helps reduce computational complexity, and a fully connected layer is introduced to predict locomotion activities. Fig 13 shows the proposed DERNN and its comparison to the previous RCNN [48]. The previous study contained input based on regression equations using laser power and scanning speed parameters; however, we proposed using the sensors and their descriptors as the input parameters based on derivatives of exponentials. The previous study utilized cube-based data formation, whereas we used slopes for each sensor-based data for further processing. Then, we sliced the data into 100 slices, and as compared to the previous method, we used DERNN instead of RCNN for training the system. The complexity of DERNN is based on the complexity of RCNN, and it is O($n^{\text{2}}$) in terms of Big O notation.

The proposed DERNN can be applied to classification problems involving dense data that require monitoring multiple parameters. For example, healthcare application systems, industrial control systems, and physical monitoring systems are a few of the potential real-time systems that may utilize the proposed model. By using DERNN, the systems can be evaluated using accuracy, precision, recall, F1 scores, and other evaluation metrics. Therefore, we can say that our work is crucial when it comes to the evaluation of locomotion prediction learning systems. This study can be further optimized using other similar methods-based comparison of DERNN with conventional deep learning methodologies.

## Results

This section describes the main findings of this proposed study along with the multiple validation methodologies. We discuss the datasets used, followed by sensor-based assessments, and provide an overall system evaluation. Furthermore, we compare the pros and cons of BSM-HMM and DERNN. A comparative study is presented to evaluate and analyze the overall performance against existing state-of-the-art systems in the literature.

### Datasets

In this subsection, a concise introduction to each dataset is presented, accompanied by a rationale for their selection in this study.

#### Opportunity++ Dataset.

Opportunity++ dataset contains data from each type of sensor, including biosensors, ambient sensors, and vision sensors. It also consists of five different sequences of activities performed during the daily living routine and a drill run. A total of seven IMU, thirteen switches, right accelerators, and an RGB video recorded at 640 × 480 resolution at 10 frames per second were included in the data. Both high-level and fine-grained level actions were performed. Hence, it is an ideal dataset for our proposed study and can be found at https://ieee-dataport.org/documents/opportunity-multimodal-dataset-video-and-wearable-object-and-ambient-sensors-based-human.

#### CMU-MMAC Dataset.

This dataset is related to kitchen and food preparation actions from daily living routines, and it was selected due to its diverse application and desirable sensor modality. It consists of five IMU, five microphones, a wearable watch, and a camera-based 4 mp resolution at 120 Hz data. A total of 55 subjects performed the preparation for brownies, sandwiches, eggs, salad, and pizza. It contains both high-level and low-level locomotor activities. It is available at http://kitchen.cs.cmu.edu/.

#### Berkeley-MHAD Dataset.

A total of 12 subjects performed eleven actions through six accelerometers, four microphones, and twelve RGB cameras. The actions that were performed are more related to the daily routine and exercise activities. Hence, it was applied to the proposed locomotion prediction system to include the flavor of physical exercise recognition. It is available at https://figshare.com/articles/dataset/Berkeley_Multimodal_Human_Action_Database_MHAD_/11809470.

#### HWU-USP Dataset.

Another dataset, HWU-USP, is included in the study to capture daily living activities, such as using a laptop, reading newspapers, using phones, etc. It contains nine such activities and a few kitchen-related actions. The data was extracted via two accelerometers, four switches, and an RGB camera of 640 × 480 at 25 frames per second rate. It is available at https://datadryad.org/stash/dataset/doi:10.5061/dryad.v6wwpzgsj.

#### LARa Dataset.

Finally, we selected a dataset of actions related to walking, pushing, pulling, carting, etc. LARa is collected through three IMU, thirty-eight infrared cameras, and an RGB camera. A total of eight actions were performed by fourteen versatile subjects in a recorded data of 840 minutes. This type of dataset has also helped to ensure the robustness of the proposed system in logistics-related actions. It is available at https://zenodo.org/records/8189341.

### Sensors-based assessment

Each sensor is evaluated separately to ensure the overall performance has met the criteria. Each type of sensor, such as biosensors, ambient, and vision sensors, has its own benefits when considered for locomotion prediction. To be certain about the overall performance of the system, we need to conduct performance validation for each sensor type that processes data separately.

#### Biosensors.

**Fig 14:**
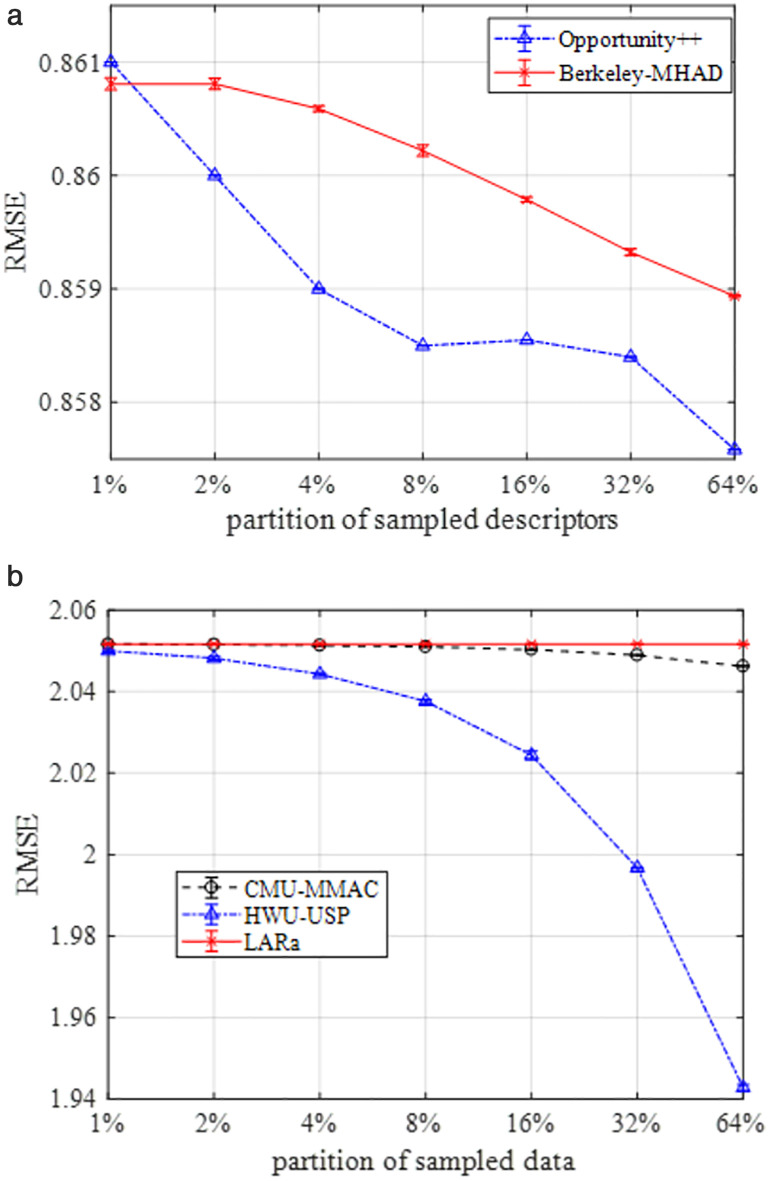
(a) RMSE-based comparison over Opportunity++ and Berkeley-MHAD; (b) RMSE-based comparison over CMU-MMAC, HWU-USP, and LARa.

A root mean square (RMSE) RMS is analyzed for the proposed system over biosensors-based data. It is calculated as (24):


$$RMS=\;\sqrt{\frac{\sum_{o=\text{1}}^{R}{(P}_{o}-A_{o}{)}^{\text{2}}}{R}}$$ (24)

where predicted outcomes $P_{o}$ and actual outcomes $A_{o}$ were used over total outcomes R. Fig 14 shows the comparison of RMSE performed over all five datasets and indicates that the RMSE declines when the partition sample data increases. In Fig 14(a), when the percentage of sampled descriptors partition is increased, the error rate for RMSE decreases significantly. However, in Fig 14(b), it is evident that increasing the sampled data partitions is not as effective over CMU-MMAC and LARa datasets. Therefore, it can be asserted that the RMSE offers a more contextually relevant measure, tailored to the specific environmental conditions of each dataset.

#### Ambient Sensors.

The interaction accuracy rate is utilized to calculate the performance of ambient sensors-based computations. The number of interactions using the upper body, hands, legs, and mid-body in each action is calculated and compared with the ground truth values given with the datasets. Tables 1–5 show the performance validation over ambient sensors-based data using the interaction accuracies over Opportunity++, CMU-MMAC, Berkeley-MHAD, LARa, and HWU-USP datasets, respectively. An average interaction accuracy rate of 96.55% across all five datasets demonstrates that the proposed system performs outstanding in computations based on ambient sensors.

**Table 1:** Ambience interaction accuracy rates (%) over Opportunity++.

Interactions	Ground Truth	Measured	Interaction Accuracy Rate (%)
^**1**^ **r**	462	452	97.83
**m**	666	637	95.64
**l**	389	370	95.12
**s**	25	24	96.00
**p**	106	98	92.45
**b**	38	36	94.74
**c**	210	205	97.62
**sp**	219	207	94.52
**Mean**			**95.50**

^1^r = reach, m = move, l = release, s = stir, p = sip, b = bite, c = cut, sp = spread.

**Table 2:** Ambience interaction accuracy rates (%) over CMU-MMAC.

Interactions	Ground Truth	Measured	Interaction Accuracy Rate (%)
^**1**^ **tk**	624	603	96.63
**p**	3866	3542	91.62
**o**	37362	37254	99.71
**f**	10113	10032	99.19
**cr**	27625	26985	97.68
**b**	4831	4706	97.41
**st**	2857	2794	97.79
**po**	3722	3710	99.68
**cl**	49663	45654	91.93
**so**	3763	3659	97.24
**re**	21333	20324	95.27
**sp**	938	927	98.83
**cs**	1066	947	88.83
**w**	1720	1683	97.85
**Mean**			96.41

^1^tk = take, p = put, o = open, f = fill, cr = crack, b = beat, st = stir, po = pour, cl = clean, so = switch on, re = read, sp = spray, cs = close, w = walk.

**Table 3:** Ambience interaction accuracy rates (%) over Berkeley-MHAD.

Interactions	Ground Truth	Measured	Interaction Accuracy Rate (%)
^**2**^ **j**	55	54	98.18
**jjk**	55	52	94.54
**be**	55	51	92.72
**pn**	55	54	98.18
**wt**	55	55	100
**wo**	55	50	90.91
**c**	55	54	98.18
**t**	55	55	100
**si**	55	55	100
**sa**	55	55	100
**sis**	55	53	96.36
**Mean**			**96.64**

^2^j = jumping, jjk = jumping jacks, be = bending, pn = punching, wt = wave two hands, wo = wave one hand, c = clapping, t = throwing, si = sit down, sa = stand up, sis = sit down/stand up.

**Table 4:** Ambience interaction accuracy rates (%) over HWU-USP.

Interactions	Ground Truth	Measured	Interaction Accuracy Rate (%)
^**5**^ **wl**	250	248	99.20
**on**	330	325	98.49
**tk**	590	587	99.49
**p**	591	581	98.31
**po**	65	62	95.38
**sr**	92	90	97.83
**sd**	32	29	90.62
**em**	16	15	93.75
**pu**	31	30	96.77
**ps**	16	16	100
**s**	48	44	91.67
**ty**	16	16	100
**m**	9	9	100
**re**	32	30	93.75
**Mean**			96.64

^5^wl = walking, on = open, tk = take, p = put, po = pour, sr = scrub, sd = stand, em = empty, pu = pull, ps = push, s = sit, ty = typing, m = move, re = reading.

**Table 5:** Ambience interaction accuracy rates (%) over LARa.

Attributes	Interactions	Ground Truth	Measured	Interaction Accuracy Rate (%)
**Legs**	^**3**^ **gc**	2421	2351	97.11
	**st**			
	**ss**			
**Hands**	**rh**	37949	37658	99.23
	**lh**			
	**nh**			
**Upper Body**	**up**	14809	13987	94.45
	**ct**			
	**dw**			
	**nim**			
	**tr**			
**Item Pose**	**bu**	1278	1270	99.37
	**hu**			
	**ua**			
	**ca**			
	**com**			
	**ni**			
**Mean**				97.54

^3^gc = gait cyle, st = step, ss = standing still, up = upwards, ct = centered, dw = downwards, nim = no intentional motion, tr = torso rotation, rh = right hand, lh = left hand, nh = no hand, bu = bulky unit, hu = handy unit, ua = utility auxillary, ca = cart, com = computer, ni = no item.

#### Vision sensors.

We suggest using a confidence level for the human body-points-based validation technique to evaluate the vision data performance. The confidence level (CL) for each human body-point can be calculated using (25) as:


$$g_{de}=\;\sqrt{\sum_{n=\text{1}}^{M}(\frac{g_{t}}{L_{c}}-\;\frac{g_{t}}{L_{c}}{)}^{\text{2}}}$$ (25)

where geodesic distance $g_{de}$ is calculated for the current location $L_{c}$ and ground truth $g_{t}$ over M values. CL supports validating the vision data performance and attaining a robust locomotion prediction system. Table 6 shows the details of each body-point along with its CL in the range [0,1] over all five datasets. An average CL of 0.94 across the datasets demonstrates that the proposed system excels in achieving acceptable performance for vision sensor-based locomotion classification. However, the CL for a few body points falls below 0.90, indicating challenges in accurately recognizing actions related to those specific body points.

**Table 6:** Confidence levels calculated for each body-point over Opportunity++, CMU-MMAC, Berkeley-MHAD, LARa, and HWU-USP.

Human Body-Points	CL for Opportunity++	CL for CMU-MMAC	CL for Berkeley-MHAD	CL for HWU-USP	CL for LARa
**head**	0.94	0.92	0.95	0.90	0.96
**neck**	0.99	0.92	0.96	0.99	0.91
**right elbow**	0.99	0.89	0.87	0.99	0.94
**right wrist**	0.97	0.99	0.99	0.98	0.93
**torso**	0.94	0.95	0.97	0.80	0.94
**left elbow**	0.94	0.97	0.89	0.97	0.98
**left knee**	0.90	0.88	0.99	0.95	0.97
**left wrist**	0.99	0.98	0.96	0.93	0.99
**right knee**	0.91	0.90	0.93	0.91	0.93
**right ankle**	0.93	0.89	0.95	0.97	0.98
**left ankle**	0.94	0.90	0.97	0.92	0.90
**Mean Confidence**	**0.95**	**0.93**	**0.95**	**0.94**	**0.95**

### Overall system evaluation

To demonstrate the performance efficiency of the proposed method, we utilize confusion matrices, accuracy rates, precision, recall, and F1-scores. Confusion matrices are particularly useful for extracting accuracy rates for each dataset and providing a detailed analysis of each activity recognition. Tables 7–11 present the accuracy rates of the locomotion prediction system for the proposed BSM-HMM across the Opportunity++, CMU-MMAC, Berkeley-MHAD, LARa, and HWU-USP datasets, respectively. Similarly, Tables 12–16 show the accuracy rates of the locomotion prediction system using the pro-posed DERNN across the same datasets.

**Table 10:** Locomotion prediction accuracy rate via confusion matrix using BSM-HMM over HWU-USP.

Locomotion	making a sandwich	tidying the kitchen	making a bowl of cereals	making a cup of tea	setting the table	using a phone	reading a newspaper	using a laptop	cleaning the dishes
**making a sandwich**	8	0	0	0	1	0	1	0	0
**tidying the kitchen**	0	8	0	0	0	1	0	1	0
**making a bowl of cereals**	1	0	7	0	1	0	0	0	1
**making a cup of tea**	0	0	1	8	0	1	0	0	0
**setting the table**	0	1	0	1	8	0	0	0	0
**using a phone**	0	0	0	0	1	7	0	1	1
**reading a newspaper**	0	0	0	0	0	0	9	0	1
**using a laptop**	1	0	1	0	0	0	0	8	0
**cleaning the dishes**	0	1	0	0	0	0	0	0	9
**Mean accuracy = 80.00%**									

**Table 11:** Locomotion prediction accuracy rate via confusion matrix using the proposed BSM-HMM over LARa.

Locomotion	standing	walking	cart	handling (upwards)	handling (centered)	handling (downwards)	Synchroni-zation	none
**standing**	7	1	0	1	0	1	0	0
**walking**	1	8	0	0	0	0	1	0
**cart**	0	0	8	0	2	0	0	0
**handling (upwards)**	0	0	1	8	0	1	0	0
**handling (centered)**	0	1	0	1	7	1	0	0
**handling (downwards)**	0	0	0	0	0	9	0	1
**synchronization**	0	0	1	0	1	0	8	0
**none**	0	0	0	0	0	1	0	9
**Mean accuracy = 80.00%**								

**Table 12:** Locomotion prediction accuracy rate via confusion matrix using the proposed DERNN over Opportunity++.

Locomotion	OP1	OP2	CL1	CL2	OPF	CLF	OD	CD	OD1	CD1	OD2	CD2	OD3	CD3	CLT	DFC	TSW
^**7**^ **OP1**	9	0	0	0	0	0	0	1	0	0	0	0	0	0	0	0	0
**OP2**	1	9	0	0	0	0	0	0	0	0	0	0	0	0	0	0	0
**CL1**	0	0	9	0	0	0	0	0	0	0	0	0	1	0	0	0	0
**CL2**	0	0	0	9	0	0	0	0	1	0	0	0	0	0	0	0	0
**OPF**	0	0	0	0	8	0	0	0	0	0	0	0	0	0	0	2	0
**CLF**	0	0	0	0	0	8	0	0	0	0	2	0	0	0	0	0	0
**OD**	0	1	0	0	0	0	7	0	0	0	0	0	0	0	2	0	0
**CD**	0	0	0	0	0	1	0	8	0	0	0	1	0	0	0	0	0
**OD1**	0	0	0	1	0	0	0	0	8	0	0	0	0	0	0	1	0
**CD1**	0	0	0	0	0	0	0	0	0	7	0	0	0	3	0	0	0
**OD2**	0	0	1	0	0	0	0	0	0	0	9	0	0	0	0	0	0
**CD2**	0	0	0	0	0	0	1	0	0	0	0	9	0	0	0	0	0
**OD3**	1	0	1	0	0	0	0	1	0	0	0	0	7	0	0	0	0
**CD3**	0	0	0	0	2	0	0	0	0	0	0	0	0	8	0	0	0
**CLT**	0	0	0	0	0	0	0	0	1	0	0	0	0	0	9	0	0
**DFC**	0	0	0	0	0	0	0	2	0	0	0	0	0	0	0	8	0
**TSW**	0	0	0	1	0	0	0	0	0	0	0	1	0	0	0	0	8
**Mean Accuracy = 82.35%**																	

^7^CD1 = Close Drawer1, OP1 = Open Door1, CL1 = Close Door1, OP2 = Open Door2, CD3 = Close Drawer3, CL2 = Close Door2, CLF = Close Fridge, OD = Open Dishwasher, CD = Close Dishwasher, OPF = Open Fridge, DFC = Drink from Cup, OD1 = Open Drawer1, CD2 = Close Drawer2, OD3 = Open Drawer3, CLT = Clean Table, OD2 = Open Drawer2, TSW = Toggle Switch.

**Table 13:** Locomotion prediction accuracy rate via confusion matrix using the proposed DERNN over CMU-MMAC.

Locomotion	stand still	walk	sit	turn	bend	kneel	stand up	sit up	sit down
**stand still**	8	1	0	0	0	0	1	0	0
**walk**	0	9	0	1	0	0	0	0	0
**sit**	0	0	9	0	0	0	0	0	1
**turn**	0	0	0	9	0	0	0	1	0
**bend**	0	0	0	0	9	1	0	0	0
**kneel**	1	0	0	0	0	9	0	0	0
**stand up**	0	0	0	0	0	0	10	0	0
**sit up**	0	0	0	0	1	0	0	9	0
**sit down**	0	0	0	0	0	0	0	0	10

Mean Accuracy = 91.11%.

**Table 14:** Locomotion prediction accuracy rate via confusion matrix using the proposed DERNN over Berkeley-MHAD.

Locomotion	jumping in place	jumping jacks	bending	punching	waving two hands	waving one hand	clapping hands	throwing a ball	sit down then stand up	sit down	stand up	T-pose
**jumping in place**	**9**	0	0	0	0	1	0	0	0	0	0	0
**jumping jacks**	0	**8**	0	0	0	0	0	1	0	1	0	0
**bending**	0	0	**9**	0	0	0	0	0	1	0	0	0
**punching**	0	1	0	**8**	0	0	1	0	0	0	0	0
**waving two hands**	0	0	1	0	**9**	0	0	0	0	0	0	0
**waving one hand**	0	0	0	0	1	**8**	0	0	0	1	0	0
**clapping hands**	0	0	0	1	0	0	**8**	0	0	0	1	0
**throwing a ball**	1	0	0	0	0	0	0	**9**	0	0	0	1
**sit down then stand up**	0	0	0	0	1	0	0	0	**9**	0	0	0
**sit down**	1	0	0	0	0	0	0	0	0	**9**	0	0
**stand up**	0	0	0	0	0	0	0	0	0	0	**9**	1
**T-pose**	0	0	0	0	0	0	0	0	0	0	0	**10**
**Mean accuracy = 87.50%**												

**Table 15:** Locomotion prediction accuracy rate via confusion matrix using the proposed DERNN over HWU-USP.

Locomotion	making a sandwich	tidying the kitchen	making a bowl of cereals	making a cup of tea	setting the table	using a phone	reading a newspaper	using a laptop	cleaning the dishes
**making a sandwich**	10	0	0	0	0	0	0	0	0
**tidying the kitchen**	0	9	0	0	0	0	1	0	0
**making a bowl of cereals**	0	0	9	0	0	0	0	0	1
**making a cup of tea**	0	0	0	9	0	1	0	0	0
**setting the table**	0	0	0	0	10	0	0	0	0
**using a phone**	0	1	0	0	0	9	0	0	0
**reading a newspaper**	0	0	0	1	1	0	8	0	0
**using a laptop**	0	0	0	0	0	0	0	10	0
**cleaning the dishes**	0	0	0	0	0	0	0	0	10
**Mean accuracy = 93.33%**									

**Table 16:** Locomotion prediction accuracy rate via confusion matrix using the proposed DERNN over LARa.

Locomotion	standing	walking	cart	handling (upwards)	handling (centered)	handling (downwards)	Synchroni-zation	none	
**standing**	9	0	0	1	0	0	0	0	
**walking**	0	8	0	0	2	0	0	0	
**cart**	0	0	8	0	0	1	0	1	
**handling (upwards)**	0	0	0	9	0	0	1	0	
**handling (centered)**	0	1	0	0	9	0	0	0	
**handling (downwards)**	0	0	0	1	0	8	1	0	
**synchronization**	1	0	1	0	0	0	8	0	
**none**	0	1	0	0	0	1	0	8	
**Mean accuracy = 83.75%**								

**Table 7:** Locomotion prediction accuracy rate via confusion matrix using the proposed BSM-HMM over Opportunity++.

Locomotion	OP1	OP2	CL1	CL2	OPF	CLF	OD	CD	OD1	CD1	OD2	CD2	OD3	CD3	CLT	DFC	TSW
^**6**^ **OP1**	8	1	0	0	0	0	0	0	1	0	0	0	0	0	0	0	0
**OP2**	0	8	0	0	1	0	0	1	0	0	0	0	0	0	0	0	0
**CL1**	0	0	8	0	0	1	0	0	0	0	0	0	0	1	0	0	0
**CL2**	1	0	1	8	0	0	0	0	0	0	0	0	0	0	0	0	0
**OPF**	0	0	0	1	9	0	0	0	0	0	0	0	0	0	0	0	0
**CLF**	0	0	0	0	0	7	1	0	0	0	1	0	0	0	1	0	0
**OD**	0	0	0	0	0	0	8	0	0	0	0	1	0	0	0	1	0
**CD**	0	0	0	0	1	0	0	7	0	1	0	0	1	0	0	0	0
**OD1**	0	0	0	0	0	0	0	0	8	0	0	0	0	0	1	0	1
**CD1**	0	1	0	0	0	0	0	0	0	9	0	0	0	0	0	1	0
**OD2**	0	0	0	0	0	0	0	0	0	0	8	0	1	0	0	1	0
**CD2**	1	0	0	0	0	0	0	0	0	0	0	9	0	0	0	0	0
**OD3**	0	0	0	0	1	0	0	1	0	0	0	0	8	0	0	0	0
**CD3**	0	0	0	0	0	0	2	0	0	0	0	2	0	6	0	0	0
**CLT**	0	1	0	0	1	0	0	0	0	0	0	0	0	1	7	0	0
**DFC**	0	0	0	0	0	1	0	0	0	0	1	0	0	0	0	8	0
**TSW**	0	0	1	0	0	0	0	0	0	0	0	0	0	0	0	0	9
**Mean Accuracy = 79.41%**																	

^6^CD1 = Close Drawer1, OP1 = Open Door1, CL1 = Close Door1, OP2 = Open Door2, CD3 = Close Drawer3, CL2 = Close Door2, CLF = Close Fridge, OD = Open Dishwasher, CD = Close Dishwasher, OPF = Open Fridge, DFC = Drink from Cup, OD1 = Open Drawer1, CD2 = Close Drawer2, OD3 = Open Drawer3, CLT = Clean Table, OD2 = Open Drawer2, TSW = Toggle Switch

**Table 8:** Locomotion prediction accuracy rate via confusion matrix using the proposed BSM-HMM over CMU-MMAC.

Locomotion	stand still	walk	sit	turn	bend	kneel	stand up	sit up	sit down
**stand still**	9	0	0	0	1	0	0	0	0
**walk**	0	10	0	0	0	0	0	0	0
**sit**	1	0	9	0	0	0	0	0	0
**turn**	0	1	0	9	0	0	0	0	0
**bend**	0	0	1	0	8	1	0	0	0
**kneel**	0	0	0	1	0	9	0	0	0
**stand up**	0	0	0	0	1	0	9	0	0
**sit up**	0	0	0	0	0	0	1	9	0
**sit down**	0	0	1	0	0	0	1	0	8
**Mean Accuracy = 88.89%**									

**Table 9:** Locomotion prediction accuracy rate via confusion matrix using the proposed BSM-HMM over Berkeley-MHAD.

Locomotion	jumping in place	jumping jacks	bending	punching	waving two hands	waving one hand	clapping hands	throwing a ball	sit down then stand up	sit down	stand up	T-pose
**jumping in place**	9	0	0	1	0	0	0	0	0	0	0	0
**jumping jacks**	0	8	0	0	0	1	0	0	0	0	0	1
**bending**	0	0	9	0	0	0	0	1	0	0	0	0
**punching**	0	1	0	7	0	0	1	0	1	0	0	0
**waving two hands**	1	0	0	0	9	0	0	0	0	0	0	0
**waving one hand**	0	0	1	0	1	8	0	0	0	0	0	0
**clapping hands**	0	0	0	1	0	0	7	0	1	0	1	0
**throwing a ball**	0	0	0	0	0	0	0	8	0	1	0	1
**sit down then stand up**	0	0	1	0	1	0	0	0	8	0	0	0
**sit down**	0	0	0	0	0	1	0	0	0	9	0	0
**stand up**	1	0	0	0	0	0	1	0	1	0	7	0
**T-pose**	0	0	0	0	0	0	0	0	0	1	0	9
**Mean accuracy = 81.67%**												

In Table 7, the proposed BSM-HMM achieves an accuracy rate of 79.41% over Opportunity++ using a machine learning-based algorithm for classification. In contrast, Table 12 shows a mean accuracy rate of 91.11% using a deep learning-based algorithm, demonstrating superior prediction performance. For the CMU-MMAC dataset, Table 8 shows a mean accuracy of 88.89% using the proposed BSM-HMM, while Table 13 shows a mean accuracy of 91.11% using DERNN, indicating that deep learning-based locomotion prediction is more accurate. Similarly, for the Berkeley-MHAD, LARa, and HWU-USP datasets, Tables 9–11 show mean accuracies of 81.67%, 80.00%, and 80.00%, respectively using BSM-HMM, while Tables 13–15 show higher mean accuracies of 87.50%, 83.75%, and 93.33% respectively using DERNN.

Based on Tables 7–16, it can be observed that the method using deep learning demonstrates somewhat superior performance in terms of accuracy rates compared to the method using machine learning.

Furthermore, precision, recall, F1-scores, and locomotion prediction accuracy rates are used to show the performance of the proposed system. (26) to (29) are utilized to calculate the precision p, recall r, F1-score f, and accuracy rates ar as:


$$p=\;\frac{trp}{trp+flp}$$ (26)

$$r=\;\frac{trp}{trp\;+fln}$$ (27)

$$f=\;\frac{\text{2}\;\times \;p\;\times \;r}{p\;+\;r}$$ (28)

$$ar=\frac{trp\;+\;trn}{trp\;+fln\;+\;trn\;+\;flp}$$ (29)

where *trp* is the true positive, *trn* is the true negative, *flp* is the false positive, and *fln* represents false negatives. Tables 17–21 demonstrate the detailed analysis using precision, recall, F1-score, and prediction accuracy rates. The comparison of mean precision, mean recall, and mean F1-scores between the BSM-HMM and DERNN classifications highlights the superiority of deep learning over the machine learning-based algorithm in our proposed methodology. The achievement of more than 0.90 results for precision, recall, and F1-scores in two datasets indicates that the system delivers outstanding results in such environments. Additionally, the other three datasets also demonstrate acceptable outcomes, achieving more than 0.80 in performance metrics.

**Table 17:** Performance evaluation for locomotion prediction using BSM-HMM and DERNN over Opportunity++ via precision, recall, F1-scores, and accuracy rate.

Opportunity++	Precision		Recall		F1-Score		Accuracy Rate	
	BSM-HMM	DERNN	BSM-HMM	DERNN	BSM-HMM	DERNN	BSM-HMM	DERNN
^**8**^ **OP1**	0.80	0.90	0.80	0.82	0.80	0.85	0.80	0.90
**OP2**	0.80	0.90	0.73	0.90	0.76	0.90	0.80	0.90
**CL1**	0.80	0.90	0.80	0.82	0.80	0.85	0.80	0.90
**CL2**	0.80	0.90	0.89	0.82	0.84	0.85	0.80	0.90
**OPF**	0.90	0.80	0.69	0.80	0.78	0.80	0.90	0.80
**CLF**	0.70	0.80	0.78	0.89	0.74	0.84	0.70	0.80
**OD**	0.80	0.70	0.73	0.87	0.76	0.77	0.80	0.70
**CD**	0.70	0.80	0.78	0.67	0.74	0.72	0.70	0.80
**OD1**	0.80	0.80	0.89	0.80	0.84	0.80	0.80	0.80
**CD1**	0.90	0.70	0.90	0.70	0.90	0.70	0.90	0.70
**OD2**	0.80	0.90	0.80	0.81	0.80	0.85	0.80	0.90
**CD2**	0.90	0.90	0.75	0.81	0.82	0.85	0.90	0.90
**OD3**	0.80	0.70	0.80	0.87	0.80	0.77	0.80	0.70
**CD3**	0.60	0.80	0.75	0.72	0.67	0.76	0.60	0.80
**CLT**	0.70	0.90	0.78	0.81	0.74	0.85	0.70	0.90
**DFC**	0.80	0.80	0.73	0.72	0.76	0.76	0.80	0.80
**TSW**	0.90	0.80	0.90	0.80	0.90	0.80	0.90	0.80
**Mean**	**0.79**	**0.82**	**0.79**	**0.80**	**0.79**	**0.81**	**0.79**	**0.82**

^8^CD1 = Close Drawer1, OP1 = Open Door1, CL1 = Close Door1, OP2 = Open Door2, CD3 = Close Drawer3, CL2 = Close Door2, CLF = Close Fridge, OD = Open Dishwasher, CD = Close Dishwasher, OPF = Open Fridge, DFC = Drink from Cup, OD1 = Open Drawer1, CD2 = Close Drawer2, OD3 = Open Drawer3, CLT = Clean Table, OD2 = Open Drawer2, TSW = Toggle Switch.

**Table 18:** Performance evaluation for locomotion prediction using BSM-HMM and DERNN over CMU-MMAC via precision, recall, F1-scores, and accuracy rate.

CMU-MMAC	Precision		Recall		F1-Score		Accuracy Rate	
	BSM-HMM	DERNN	BSM-HMM	DERNN	BSM-HMM	DERNN	BSM-HMM	DERNN
**stand still**	0.90	0.80	0.90	0.89	0.90	0.84	0.90	0.80
**walk**	1.00	0.90	0.91	0.90	0.95	0.90	1.00	0.90
**sit**	0.90	0.90	0.82	1.00	0.86	0.94	0.90	0.90
**turn**	0.90	0.90	0.90	0.90	0.90	0.90	0.90	0.90
**bend**	0.80	0.90	0.80	0.90	0.80	0.90	0.80	0.90
**kneel**	0.90	0.90	0.90	0.90	0.90	0.90	0.90	0.90
**stand up**	0.90	1.00	0.82	0.90	0.86	0.94	0.90	1.00
**sit up**	0.90	0.90	1.00	0.90	0.95	0.90	0.90	0.90
**sit down**	0.80	1.00	1.00	0.90	0.89	0.94	0.80	1.00
**Mean**	**0.89**	**0.91**	**0.89**	**0.91**	**0.89**	**0.90**	**0.89**	**0.91**

**Table 19:** Performance evaluation for locomotion prediction using BSM-HMM and DERNN over Berkeley-MHAD via precision, recall, F1-scores, and accuracy rate.

Berkeley- MHAD	Precision		Recall		F1-Score		Accuracy Rate	
	BSM-HMM	DERNN	BSM-HMM	DERNN	BSM-HMM	DERNN	BSM-HMM	DERNN
**jumping in place**	0.90	0.90	0.81	0.81	0.85	0.85	0.90	0.90
**jumping jacks**	0.80	0.80	0.88	0.89	0.84	0.84	0.80	0.80
**bending**	0.90	0.90	0.82	0.90	0.86	0.90	0.90	0.90
**punching**	0.70	0.80	0.77	0.89	0.73	0.84	0.70	0.80
**waving two hands**	0.90	0.90	0.88	0.81	0.84	0.85	0.90	0.90
**waving one hand**	0.80	0.80	0.80	0.89	0.80	0.84	0.80	0.80
**clapping hands**	0.70	0.80	0.78	0.89	0.74	0.84	0.70	0.80
**throwing a ball**	0.80	0.90	0.89	0.90	0.84	0.90	0.80	0.90
**sit down then stand up**	0.80	0.90	0.72	0.90	0.76	0.90	0.80	0.90
**sit down**	0.90	0.90	0.82	0.81	0.85	0.85	0.90	0.90
**stand up**	0.70	0.90	0.87	0.90	0.78	0.90	0.70	0.90
**T-pose**	0.90	1.00	0.82	0.83	0.85	0.90	0.90	1.00
**Mean**	**0.81**	**0.87**	**0.82**	**0.86**	**0.81**	**0.86**	**0.82**	**0.87**

**Table 20:** Performance evaluation for locomotion prediction using BSM-HMM and DERNN over HWU-USP via precision, recall, F1-scores, and accuracy rate.

HWU-USP	Precision		Recall		F1-Score		Accuracy Rate	
	BSM-HMM	DERNN	BSM-HMM	DERNN	BSM-HMM	DERNN	BSM-HMM	DERNN
**making a sandwich**	0.80	1.00	0.80	1.00	0.80	1.00	0.80	1.00
**tidying the kitchen**	0.80	0.90	0.80	0.90	0.80	0.90	0.80	0.90
**making a bowl of cereals**	0.70	0.90	0.78	1.00	0.73	0.94	0.70	0.90
**making a cup of tea**	0.80	0.90	0.88	0.90	0.84	0.90	0.80	0.90
**setting the table**	0.80	1.00	0.72	0.90	0.76	0.94	0.80	1.00
**using a phone**	0.70	0.90	0.78	0.90	0.74	0.90	0.70	0.90
**reading a newspaper**	0.90	0.80	0.90	0.89	0.90	0.84	0.90	0.80
**using a laptop**	0.80	1.00	0.80	1.00	0.80	1.00	0.80	1.00
**cleaning the dishes**	0.90	1.00	0.75	0.90	0.82	0.94	0.90	1.00
**Mean**	**0.80**	**0.93**	**0.80**	**0.93**	**0.79**	**0.92**	**0.80**	**0.93**

**Table 21:** Performance evaluation for locomotion prediction using BSM-HMM and DERNN over LARa via precision, recall, F1-scores, and accuracy rate.

LARa	Precision		Recall		F1-Score		Accuracy Rate	
	BSM-HMM	DERNN	BSM-HMM	DERNN	BSM-HMM	DERNN	BSM-HMM	DERNN
**standing**	0.70	0.90	0.87	0.90	0.78	0.90	0.70	0.90
**walking**	0.80	0.80	0.80	0.80	0.80	0.80	0.80	0.80
**cart**	0.80	0.80	0.80	0.89	0.80	0.84	0.80	0.80
**handling (upwards)**	0.80	0.90	0.89	0.81	0.84	0.85	0.80	0.90
**handling (centered)**	0.70	0.90	0.70	0.81	0.70	0.85	0.70	0.90
**handling (downwards)**	0.90	0.80	0.69	0.80	0.78	0.80	0.90	0.80
**synchronization**	0.80	0.80	0.89	0.80	0.84	0.80	0.80	0.80
**none**	0.90	0.80	0.90	0.89	0.90	0.84	0.90	0.80
**Mean**	**0.80**	**0.83**	**0.82**	**0.83**	**0.80**	**0.83**	**0.80**	**0.83**

### Performance Comparison between BSM-HMM and DERNN

The proposed method can have either the BSM-HMM as a machine learning-based classifier or DERNN as a deep learning-based classifier. To compare the performance of each method over the locomotion prediction system, we compared the accuracy rates and computational times over all five datasets for both approaches. Table 22 provides a detailed performance comparison according to the computational times and prediction accuracies. From the comparison, we suggest that using BSM-HMM is favorable when computational time is more important than the accuracy rate. However, the DERNN-based method is more acceptable when the system performance in terms of accuracy is more critical.

**Table 22:** Performance comparison of BSM-HMM and DERNN techniques using mean computational time and mean accuracy rates (%).

Datasets	Opportunity++	CMU-MMAC	Berkeley-MHAD	LARa	HWU-USP	Mean
**Mean computational time (s)**						
**DERNN**	256	249	236	240	251	246.4
**BSM-HMM**	213	208	196	199	211	205.4
**Mean accuracy rates (%)**						
**DERNN**	82.35	91.11	87.50	83.75	93.33	87.61
**BSM-HMM**	79.41	88.89	81.67	80.00	80.00	81.99

### Comparison of the proposed system with other methods

Other existing methods for locomotion prediction utilize activity recognition for daily living actions. In contrast, our proposed system has achieved a mean accuracy rate of 87.61%, which is improved than the systems compared in literature. This was achieved through the customized techniques developed in this study for data filtering, descriptor extraction, descriptor selection, and classification. We have compared the proposed system with previous studies, and Table 23 presents a comprehensive comparison. As shown in Table 23, the proposed method using BSM-HMM achieves slightly better performance. However, significantly better results can be achieved when using DERNN in the performance evaluation based on the accuracy of locomotion activity predictions.

**Table 23:** A comparison between the proposed method using BSM-HMM and DERNN and previous works has been given using accuracy rates (%).

Methods	Opportunity++	CMU-MMAC	Berkeley-MHAD	LARa	HWU-USP
Javeed et al. [12]	74.15	–	–	–	–
Azmat et al. [49]	74.70	–	–	–	–
Akhter et al. [50]	75.29	–	–	–	–
Lu et al. [51]	–	86.60	–	–	–
Batool et al. [52]	–	88.00	–	–	–
Hafeez et al. [53]	–	89.50	–	–	–
Lannan et al. [54]	–	–	83.92	–	–
Tian et al. [55]	–	–	84.00	–	–
Lannan et al. [56]	–	–	84.00	–	–
Lüdtke et al. [57]	–	–	–	71.50	–
Awasti et al. [58]	–	–	–	75.90	–
Syed et al. [59]	–	–	–	78.61	–
Javeed et al. [9]	–	–	–	–	82.22
Javeed et al. [60]	–	–	–	–	87.67
Ranieri et al. [29]	–	–	–	–	92.33
**Proposed BSM-HMM**	**79.41**	**88.89**	**81.67**	**80.00**	**80.00**
**Proposed DERNN**	**91.11**	**88.89**	**87.50**	**93.33**	**83.75**

In previous studies, several approaches have been proposed for human motion recognition and locomotion prediction; however, many of these methods exhibited limitations in feature extraction, pre-processing, and classification. For instance, [12] introduced a four-module framework involving signal pre-processing, segmentation, feature extraction, and feed-forward neural network-based classification. Nonetheless, the system underperformed due to insufficient feature extraction and suboptimal classifiers. Similarly, [49] proposed an IoT-based data processing system for home surveillance, yet it suffered from irrelevant feature selection and ineffective discrimination techniques, resulting in low accuracy. In [50], a deep belief network was utilized for skeleton modeling based on multi-sensor data, but ineffective filtration allowed noise interference, impairing motion recognition performance.

Advanced neural architectures have also been explored to address these challenges. For example, [51] combined a recurrent Capsule Network (CapsNet) with a ConvLSTM to capture spatio-temporal features, while optimizing parameters using a genetic algorithm. However, the reliance on hand-crafted features and a lack of pre-processing led to limited success. Similarly, Batool et al. [52] implemented data filtration, feature extraction, optimization, and classification to monitor daily activities. Despite using a reweighted genetic algorithm and noise removal, the system struggled to detect complex actions. In [53], sophisticated cue extraction methods, such as Hilbert and Walsh-Hadamard transforms, Bone Pair Descriptors, waypoint trajectories, and random occupancy patterns, were adopted. Yet, the absence of optimal cue selection contributed to degraded accuracy.

Several other studies further illustrate similar shortcomings. In [54], Tobit Kalman filtering and convolutional autoencoders were applied for motion capture, though accuracy levels remained unsatisfactory. A multi-model learning approach in [55] utilizing AlexNet, LSTM, BiLSTM, LeNet, and ResNet achieved recognition accuracy below 84%. Additionally, the skeleton generation and matching technique in [56] failed to deliver acceptable performance due to a lack of pre-processing and feature reduction. A hybrid attribute-based deep neural network in [57] showed inefficiency in recognizing human actions owing to unfiltered sensor data. Likewise, transfer learning-based pose estimation in [58] and the inefficient filtration strategy in [59] led to subpar results.

In more recent studies, [9] leveraged data filtration, state-of-the-art descriptor extraction, and a residual neural network for classification, but insufficient descriptor optimization reduced detection accuracy. The system proposed in [60] integrated pre-processing, feature engineering, fusion, optimization, and classification for human action detection, yet the performance was limited due to ineffective feature engineering. Finally, [29] applied LSTM and CNN models for action prediction and classification, but the lack of comprehensive pre-processing and feature extraction led to unsatisfactory outcomes.

### Ablation Study

This study has proposed a locomotion prediction system for practical implementation, utilizing multi-sensory devices and ontological agents. The system efficiency stems from its novel approach to locomotion prediction through innovative filtration, descriptor extraction, and customized classification methodologies. While experimental outcomes demonstrate the robustness and accuracy of the proposed system, an ablation study is conducted to further clarify its competence and utility.

Table 24 shows the effectiveness of the proposed system with and without the filtration method, proposed descriptor extraction techniques, and DERNN. The comparative analysis employs accuracy rates, peak signal-to-noise ratio (PSNR), and mean squared error (MSE) across a diverse range of applications [59]. The results indicate that the performance of the proposed system is enhanced through the implementation of these novel approaches.

**Table 24:** Effectiveness of our proposed locomotion prediction system has been performed using accuracy rate (%), peak signal-to-noise ratio (PSNR), and mean squared error (MSE).

Performance Parameters	Opportunity++	CMU-MMAC	Berkeley-MHAD	LARa	HWU-USP
without novel filter, descriptors, and DERNN					
Accuracy rate (%)	70.45	79.65	72.97	80.50	69.84
PSNR (dB)	34.56	35.67	33.21	30.47	29.18
MSE	55.65	60.32	58.94	62.84	55.21
with proposed filter, descriptors, and DERNN					
Accuracy rate (%)	82.35	91.11	87.50	93.33	83.75
PSNR (dB)	49.21	48.54	46.21	48.60	43.58
MSE	2.55	1.99	2.99	2.47	3.14

### Limitations of the study

**Fig 15:**
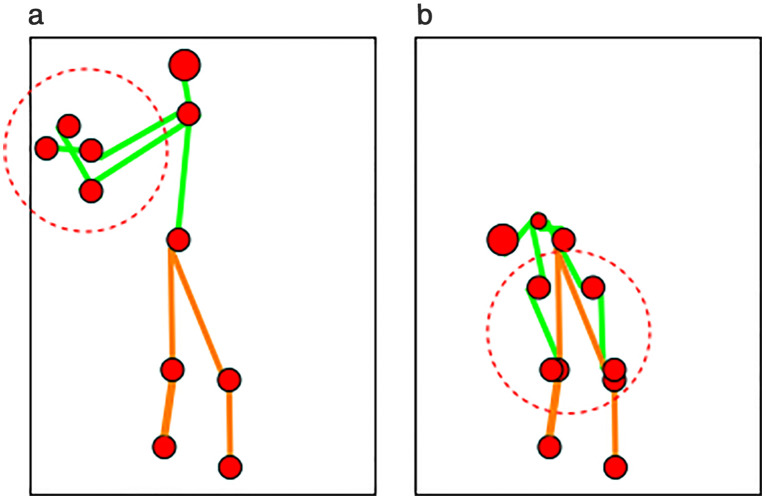
(a) Mixed up body points for left wrist and right wrist shown in red dashed circle; (b) Mixed up skeleton body points for left wrist and left knee along with right wrist and right knee in red dashed circle.

Despite of achieving improved accuracy rates and other matricular achievements in this study, there are some challenges present in the system that need to be looked into for future work. The system is limited when it comes to identifying correct body points for human skeleton modeling due to the complex human actions present in daily living routines causing pose estimation limitation. For example, Fig 15a and 15b shows the red circled body points that can be mixed up together with each other due to random human motion actions like daily workout routines. If we look at the MSE and accuracy rates in Table 24, we can see that this limitation has contributed towards higher MSE in prediction of actual true activities causing the performance degradation in terms of accuracy rates. Although the accuracy rates have been affected by this limitation, but due to multi-sensors-based approach, we were able to achieve better accuracies when compared to literature as shown in Table 23. Table 25 gives a detailed insight into the experiment performed using the confused human actions by providing a focused confusion matrix for those activities that were mixed together by system over Berkeley-MHAD dataset. For example, we can see from the table that clapping hands action has been confused with jumping jacks, punching, and waving one hand actions. Similarly, jumping jacks action was confused with punching and waving one hand actions. Such confusions have caused degraded performance of the proposed system. In future, a more detailed analysis of such complex motion patterns needs to be done, e.g., human tracking or distinguishing movement patterns strategies can be applied in order to avoid degraded accuracy due to these failure cases and increase the practical use of our proposed system.

**Table 25:** Locomotion prediction focused confusion matrix using the proposed DERNN over Berkeley-MHAD.

Locomotion	jumping jacks	punching	waving one hand	clapping hands
**jumping jacks**	**8**	0	1	1
**punching**	1	**8**	0	1
**waving one hand**	1	0	**8**	1
**clapping hands**	0	1	1	**8**

## Conclusions

This study presents a novel adaptive system for locomotion prediction that demonstrates robustness across various environmental conditions and sensor-derived data inputs. The key contributions of this research, namely the innovative data filtration technique, advanced descriptor extractors and selectors, and sophisticated motion classifiers, have collectively enhanced the system’s performance to optimal levels. We introduced a versatile locomotion prediction system with potential applications in domains such as smart homes, healthcare, surveillance, and lifelogging. The success of the proposed approach is underpinned by the integration of machine learning, ontological agents, deep learning, graph theory, filtration mathematics, semantic relations, and a combination of sensor data.

However, the system faces challenges, particularly in accurately identifying body points for human skeleton modeling, which has resulted in a reduction in overall performance. This has been reflected in Fig 15 of the previous section. To address these limitations, future work will incorporate advanced techniques such as MediaPipe or YOLO-V8 and integrate additional enhancements to improve the capabilities of intelligent agents and optimize the system’s overall performance.
